# Consensus on the clinical utility of digital mobility outcomes for personalized clinical decision support in parkinson’s disease

**DOI:** 10.1186/s42466-025-00426-8

**Published:** 2025-09-18

**Authors:** Alan Castro Mejia, Stefano Sapienza, Ivana Paccoud, Lisa Alcock, Philip Brown, Heiko Gaßner, Heather Hunter, Walter Maetzler, Anat Mirelman, Alice Nieuwboer, Martin Regensburger, Lynn Rochester, Sabine Stallforth, Beatrix Vereijken, Alison Yarnall, Jochen Klucken

**Affiliations:** 1 Luxembourg Centre for Systems Biomedicine, Digital Medicine Group, University of Luxembourg, Esch-sur-Alzette, Luxembourg; 2 Centre Hospitalier de Luxembourg, Luxembourg City, Luxembourg; 3 NIHR Newcastle Biomedical Research Centre, Newcastle University, Campus for Ageing and Vitality, Newcastle upon Tyne, UK; 4 The Newcastle upon Tyne Hospitals NHS Foundation Trust, Newcastle upon Tyne, UK; 5 Department of Molecular Neurology, Friedrich-Alexander-Universität Erlangen-Nürnberg, Erlangen, Germany; 6 Translational and Clinical Research Institute, Faculty of Medical Sciences, Newcastle University, Newcastle upon Tyne, UK; 7 Department of Neuromedicine and Movement Science, Norwegian University of Science and Technology, Trondheim, Norway; 8 Department of Neurology, University Hospital Schleswig-Holstein and Kiel University, Kiel, Germany; 9 Laboratory for Early Markers of Neurodegeneration (LEMON), Tel Aviv Sourasky Medical Center, Tel Aviv, Israel; 10 School of Medicine & Health Sciences, Tel Aviv University, Tel Aviv, Israel; 11 Sagol School of Neuroscience, Tel Aviv University, Tel Aviv, Israel; 12 Fraunhofer Institute for Integrated Circuits IIS, Erlangen, Germany; 13 Neurorehabilitation Research Group (eNRGy), Department of Rehabilitation Sciences, KU Leuven, Leuven, Vlaams-Brabant Belgium; 14 Leuven Brain Institute (LBI), Leuven, Belgium

**Keywords:** Digital mobility outcomes, Parkinson’s disease, Gait, Consensus study, Clinical utility

## Abstract

**Background:**

Digital mobility outcomes (DMOs) have emerged as novel biomarkers offering objective, quantitative, and examiner-independent outcome measures for clinical studies. Unfortunately, research efforts on DMOs have not yet investigated the domain of clinical utility in Parkinson’s disease, i.e. providing evidence of improvements in health outcomes, diagnosis, decision-making, or prevention when compared to e.g. standard-of-care procedures. This manuscript, via a consensus building approach, aims to create a structured conceptual framework to map the knowledge generated by DMOs with clinical domains that could benefit from it.

**Methods:**

We conducted a three-round consensus-building study with 12 experts recruited from the Mobilise-D consortium’s Parkinson’s Disease Working Group. The experts designed and ranked different aspects of the conceptual framework via a 5-level Likert scale for level of agreement. Consensus for the different points evaluated was based on a double threshold: the simultaneous presence of a high level of agreement had to be accompanied by a low level of disagreement. As secondary objectives, the experts were asked to rate the practical application of DMOs by evaluating the timeline to applicability, the foreseen challenges for their implementation in clinical settings, and their main role in the decision-making process.

**Results:**

A full consensus on the clinical utility framework was achieved after three rounds. The final framework consisted of three main categories (*Disease Diagnosis*, *Patient Evaluation*, and *Treatment Evaluation*) and six underlying domains (*Enhancing Diagnostic Procedure*, *Predicting Risk*, *Timely Detecting Deterioration*, *Enhancing Clinical Judgment*, *Selecting Treatment*, and *Monitoring Treatment Response*). The experts believed in the next 1–5 years DMOs will play a relevant role in clinical decision making, complementing care knowledge with useful digital biomarkers information. However, the main challenge to address is the definition of clear reference value for DMOs interpretability.

**Conclusions:**

This framework provides a structure for subsequent studies to build into by diversifying expert cohorts and expand our findings beyond PD. Additionally, our results support researchers planning future clinical trials where DMOs can play a valuable role for clinical decision support. Ultimately, this is the first step toward developing guidelines to assess DMOs’ clinical utility and support their integration into Real World clinical practice.

**Supplementary Information:**

The online version contains supplementary material available at 10.1186/s42466-025-00426-8.

## Background

Mobility, the ability to move freely and easily [1], is a primary constituent of a healthy life, with speed widely considered the sixth vital sign [2]. In patients with Parkinson’s disease (PD) who are affected by chronic motor disorders that progress and fluctuate over time, the characterization of mobility bears critical importance for the assessment of overall conditions and subsequent management of treatment. Due to the extreme heterogeneity of clinical presentations in PD, interventions and clinical decisions must be tailored to the patient’s characteristics [3]. This practice, defined as personalized care, requires an accurate and comprehensive evaluation of the patient’s profile. In this context, mobility-related information assumes a primary role.

Digital mobility outcomes (DMOs) have emerged as novel biomarkers offering objective, quantitative, and examiner-independent outcome measures for clinical studies [1, 4]. Unlike traditional clinical assessments, which are often subjective and vary depending on the examiner’s expertise, DMOs provide a standardized approach to capture real-world mobility through digital technologies, such as wearable devices. As such, DMOs are gaining recognition as reliable tools for characterizing patient status, evaluating treatment responses, and supporting regulatory decision-making across various medical conditions [5]. For PD in particular, several studies, including those from the Mobilise-D consortium, have highlighted the ability of DMOs to provide objective, relevant, and validated information that characterizes both symptom severity and progression rates [1, 6–11]. Additionally, promising examples of the clinical applicability of DMOs show that these may differentiate across PD and non-PD groups [12], medication states, targeted gait outcomes (e.g., to detect freezing events) [13], correlate with classical clinical scales such as the MDS-UPDRS [14], and offer clinically meaningful measures of mobility after rehabilitation for long-term assessment [15].

Furthermore, DMOs have gained recognition as endpoints for clinical studies focusing on mobility [16, 17]. The reason is twofold: the necessity of detecting subtle gait changes that cannot be visually captured or quantified and the possibility of extending the monitoring window outside the clinic. This scientific evidence suggests that DMOs should represent an essential tool for clinicians and healthcare professionals.

Unfortunately, research efforts on DMOs have not yet investigated the domain of clinical utility, providing evidence of improvements in health outcomes, diagnosis, decision-making, or prevention when compared to standard-of-care procedures. A recent literature review focusing inertial sensors for home monitoring in PD showed that only 15% of studies provided evidence on clinical utility [18], with most presenting findings in qualitative terms. Although DMOs have been touted as playing a complementary role in enhancing current standards of care by supporting clinical decision-making and personalized intervention strategies [6], the lack of clear reference values and difficulty in integrating results into care pathways [6, 19] have hindered their translation from validated technical measures to decision support tools within the clinical management of PD. A myriad of studies demonstrated the technical accuracy of DMOs, and their correlation with standard clinical scales. However, to generate clinical utility, DMO information must be conveyed into actionable items that physicians can apply to individual patient cases. This task involves translating results from highly structured clinical and research studies through the lens of noisy real-world scenarios. Thus, a key first step toward resolving this issue would be to map the knowledge generated by DMOs with clinical domains that could benefit from it.

A structured conceptual framework may bridge the translational gap between research advancements and clinical workflows, encouraging future studies to demonstrate and provide evidence of clinical utility. Therefore, as a primary objective, this expert consensus manuscript aims to build on the clinical and technical knowledge from the Mobilise-D consortium to define the clinical domains where DMOs could be implemented to support personalized care in PD. As a secondary objective, the study seeks to gather expert perspectives on the practical implementation. Ultimately, the goal is to facilitate the translation from *research knowledge* to *clinical utility* of this new information generated by DMOs.

## Methods

We conducted a three-round consensus-building study between April 9 and November 27, 2024, to support the integration of DMOs in clinical workflows in PD. As empirical data is unavailable, we chose an electronic-based consensus-building methodology. This method allowed for the development of guidance on the basis of iterative rounds of discussion, voting, and refinement until a predefined expert consensus threshold was reached [20, 21]. Additionally, the electronic format allowed for asynchronous feedback and added flexibility to ensure the participation of an international expert panel [21].

### Expert panel

A single expert panel was recruited from the Mobilise-D consortium’s Parkinson’s Disease Working Group [10]. This project included 12 international academics with diverse healthcare expertise in PD. Site leaders disseminated the project internally, and interested individuals received a formal enrollment invitation detailing the aim, objectives, and project methodology. Implicit consent was provided by accepting the invitation and voting anonymity was preserved to ensure equal treatment of expertise across the panel.

### Consensus-building procedure

Before each round, the experts received an overview of the goals, including the rating scale and the consensus criteria. All materials provided to the experts were piloted internally with clinicians, clinical researchers, social scientists, and biomedical engineers.

The experts ranked different aspects of a conceptual framework via a 5-level Likert scale for level of agreement *(1 = Strongly Disagree*, *2 = Disagree*, *3 = Neither Agree nor Disagree*, *4 = Agree*, *and 5 = Strongly Agree).* A 5-level scale is a robust metric against rounding errors. It measures the intensity of the rater’s opinion depending on their level of agreement or disagreement (e.g., “*Agree*” vs. “*Strongly Agree*”) [22]. Additionally, an open-ended question was provided at the end of each voting section to gather further expert feedback.

Consensus for the different points evaluated was based on a double threshold: the simultaneous presence of a high level of agreement had to be accompanied by a low level of disagreement. Consensus was thus defined as an average agreement across the experts equal to or above level 4 (i.e., “*Agree*”), with the simultaneous absence of disagreement (levels 1 and 2). Similar methods have been used in predefined consensus-finding studies to ensure adequate representation of disagreements, even those from minority groups [23–28]. In case of disagreement, experts were asked to describe their concerns in free text and provide insights into the reasons for their disagreement. This feedback was utilized in synergy with the overall agreement to update the framework iteratively.

#### Initial framework

**Fig. 1:**
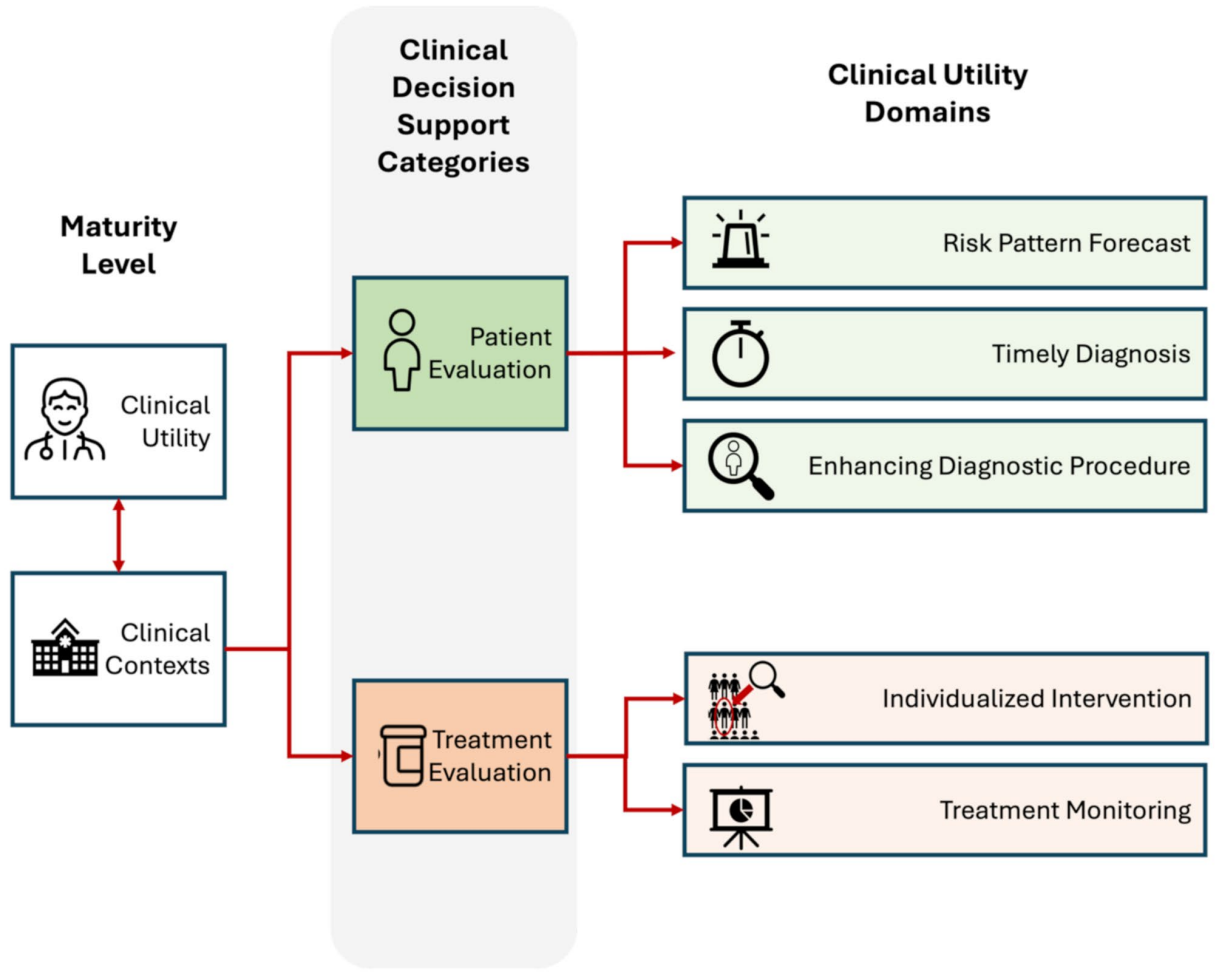
Predefined Framework based on the treatment cycle concept where the patient management strategy is considered composed by periodic, alternating diagnostic and treatment periods herein represented as *Clinical Decision Support Categories*. The categories are subsequently divided into domains according to the value DMOs can provide. This has been the initial framework presented to the expert panel in round 1

As a starting point for the consensus study, we predefined a framework based on the “treatment cycle” concept described by Klucken et al. [29], where the progression of patient disease is segmented by alternating phases of patient and treatment evaluation. These phases, denominated categories, converge at the clinical consultation, where the physician goes from *(a)* assessing risks, symptoms, or diseases to *(b)* selecting and monitoring the treatment. The primary categories were then subdivided into five secondary domains where DMOs may provide value to the clinical team and thus achieve clinical utility (Fig. 1). For the present study, clinical utility was defined as improving health outcomes, diagnosis, treatment management, or prevention after the use of DMOs [18]. Notably, domains represent distinct clinical contexts and hold different requirements for digital outcomes to provide value. Examples of clinical contexts and potential values were provided for each domain to ensure adequate interpretability.

#### Rounds 1 and 2

The primary objective of the first round was to find consensus on the established conceptual framework. The experts reviewed a prerecorded workshop describing the “treatment cycles” concept along with the corresponding framework. Then, the experts voted on the overall structure and concept, the contexts, and the clinical values of the DMOs through the 5-level Likert scale described above. The round closed with open-ended suggestions for changes to the framework, including missing domains, updates to the values, or changes to the naming conventions.

#### Round 3

The primary objective of the third and final round was to review the final framework and present the final structure, taxonomy, and definitions derived from the previous rounds. In contrast to the previous rounds, this final stage was an online interactive session intended to clarify any remaining points from the final framework, allowing for quicker iteration of feedback and sign-off on the study results. Items requiring voting remained anonymous and followed the double-threshold technique as previously employed.

#### Secondary study objectives

We sought expert opinions on three secondary items about the practical application of DMOs by evaluating the timeline to applicability, the challenges foreseen for their implementation in clinical settings, and their main role in the decision-making process. The experts assessed the first two through a single-answer, six-item scale (*currently applicable*, *1 to 5 years*, *5 to 10 years*, *not clinically applicable*, *I do not know*). The third one was evaluated through a multiple choice single-answer question.

### Data analysis

Averages were calculated for each voting item. The open-ended questions were grouped by theme and assessed independently of the Likert scale responses. Finally, the participation rate was computed on the basis of the number of enrolled participants and the number of responses received within the allotted time. Descriptive statistics were calculated using a spreadsheet processor. No imputation was performed for the replies of the experts who dropped out from the study.

## Results

Twelve experts from six academic institutions across Europe received invitations to participate, including 10 (83%) clinical and 2 (17%) technical experts with expertise in neurology, geriatrics, cognition, physiotherapy, biomechanics, and gait analysis (Table 1).

**Table 1:** Panel expert characteristics. Results are expressed as “mean (Standard Deviation)” or “Unit, Percentage” according to the variable considered

Characteristics	All Panel Experts (*n* = 12)	Clinical Background (*n* = 10)	Technical Background (*n* = 2)
**Average Age**, **years**	50.33 (9.54)	50.60 (9.16)	49.00 (7.24)
**Sex (*n***, **%)**			
• Male • Female	4, 33% 8, 67%	4, 40% 6, 60%	0, 0% 2, 100%
**Education (***n*, **%)**			
• Medicine • Psychology • Physiotherapy • Sports Science	4 1 5 2	4 0 5 1	0, 0% 1, 50% 0, 0% 1, 50%
**Background (***n*, **%)**			
• Clinical • Technical	10, 83% 2, 17%	10, 100% 0, 0%	0, 0% 2, 100%
**Residence (*n***, **%)**			
• United Kingdom • Germany • Belgium • Norway • Israel	5, 42% 4, 33% 1, 8% 1, 8% 1, 8%	4, 40% 4, 40% 1, 10% 0, 0% 1, 10%	1, 50% 0, 0% 0, 0% 1, 50% 0, 0%
**Average Experience**, **years**	16.75 (7.24)	17.40 (15.56)	13.50 (2.12)

### Round 1 results

**Fig. 2:**
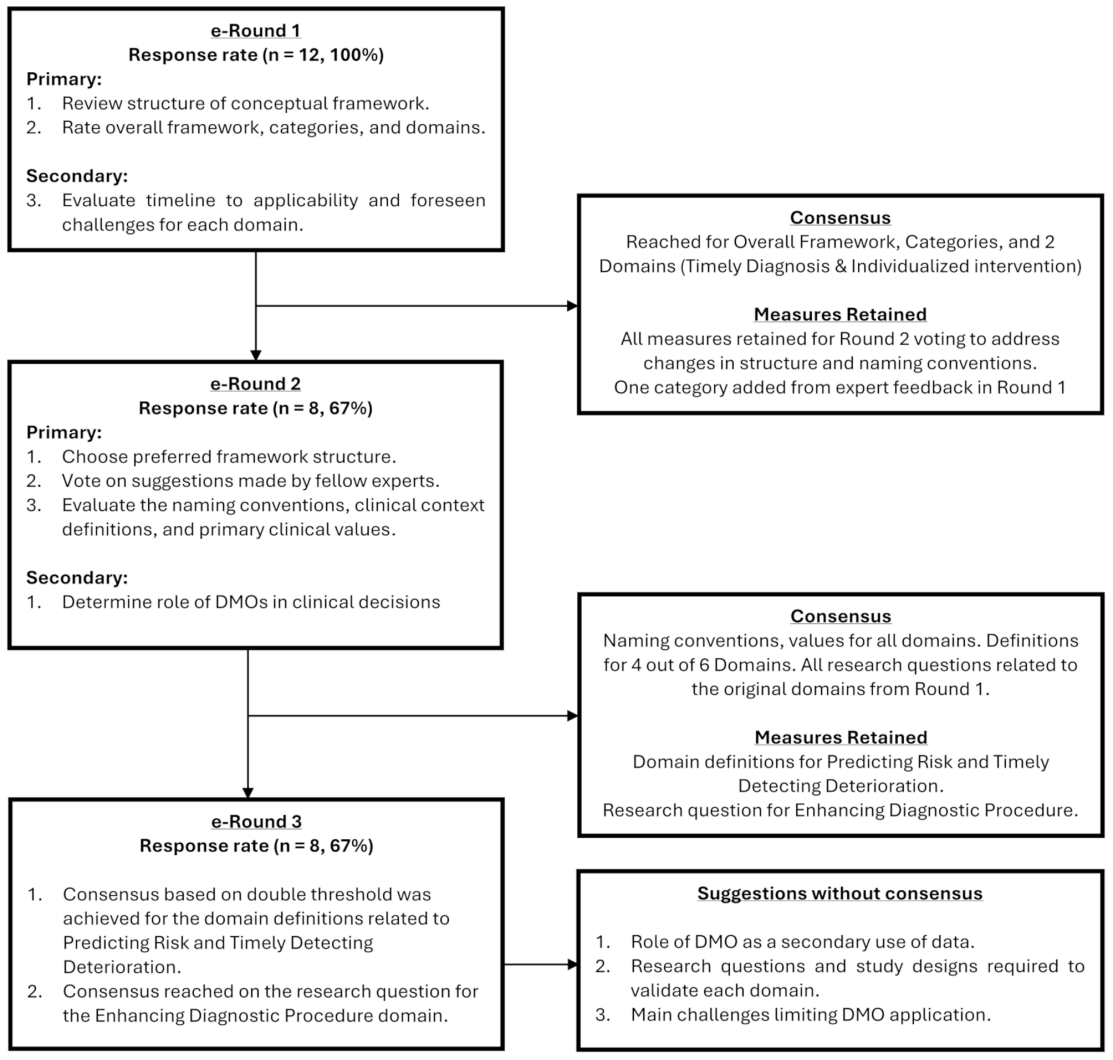
Flow diagram of the modified consensus methodology. The figure describes the primary and secondary objectives targeted during each round, and the consensus achieved in each iteration

Round 1 had a 100% response rate (Fig. 2). The experts reached a consensus across the overall framework concept, the two categories, and the *Timely Diagnosis* and *Individualized Intervention* domains (Table S1). From a conceptual applicability standpoint, *Individualized Intervention* was the highest-rated domain, with an overall agreement of 4.64, followed by *Timely Diagnosis* (4.55) and *Treatment Monitoring* (4.45) (Table S3). Additionally, *Timely Diagnosis (4.82) and Enhancing Diagnostic Power (4.36)* received the most approval among the examples evaluating clinical contexts and values (Table. S2-S3).

The most common elements of disagreements in this round were associated with two domains: *Risk Pattern Forecast* and *Treatment Evaluation*. Panel experts suggested changing the naming convention of *Risk Pattern Forecast*, defining the term “individualized,” and narrowing the scope of each domain to an actionable clinical process. Experts expressed concerns regarding the prognostic power of DMOs, which were seen as more capable of detecting subtle deterioration rather than being hallmarks of future impairment. Updates to the *Treatment Evaluation* domains were also requested to account for treatment feedback loops and patient self-management. All measures were retained for voting in Round 2 to ensure alignment with changes derived from the feedback provided. Finally, experts requested that disease diagnosis be treated as a separate category and not be embedded within the *Patient Evaluation*.

Concept-related suggestions were split into taxonomy or definition comments and explored separately in the subsequent rounds.

### Round 2 results

Round 2 had a lower response rate of 67%, with 8 of the original 12 experts responding. Based on Round 1 feedback, two framework structures were presented to the experts (Fig. S4). 75% of experts voted for a new *Disease Diagnosis* category and corresponding domain to be added to the framework (Fig. S4a) rather than having it nested within the *Patient Evaluation* category. The remaining two experts preferred an updated version of the original structure presented in Round 1 without a new category (Fig. S4b). However, one of the two experts provided open feedback that aligned with the new category structure, increasing the acceptance of the additional category to 87.5%. All other inclusions to the framework were rejected.

Independent of the framework’s structure, the experts reached a consensus on the taxonomy, values, and 4 out of 6 domain definitions (Table 2). Additionally, consensus was reached on most research questions, apart from *Enhancing Diagnostic Procedure*, which was not tested as it was the domain corresponding to the new category.

**Table 2:** Expert consensus results by round. ªThese symbols denote absence of double threshold consensus. Check (✓) denotes consensus reached. *Denotes averages calculated over 6 experts only due to conceptual differences between V1 and V2 framework

Expert Consensus on Framework Domains	Average Agreement Results							
	Round 1		Round 2			Round 3		
	Concept	Clinical Value	Concept		Clinical Value	Concept		Clinical Value
			Taxonomy	Definition		Taxonomy	Definition	
Enhancing Diagnostic Procedure	-	-	4.50*	4.33*	4.00*	✓	✓	✓
Predicting Risk	4.36ª	4.09ª	4.50*	3.67*ª	4.17*	✓	4.38	✓
Detecting Timely Deterioration	4.82ª	4.45ª	4.38	4.25ª	4.50	✓	4.38	✓
Enhancing Symptom Assessment	4.36ª	4.09ª	4.50	4.50	4.50	✓	✓	✓
Selecting Treatment	4.00ª	3.73ª	4.50	4.38	4.25	✓	✓	✓
Monitoring Treatment Response	4.27ª	4.18ª	4.63	4.00	4.25	✓	✓	✓

The main disagreements were associated with the domains of *Predicting Risk* and *Detecting Timely Deterioration*. In particular, one expert disagreed with the terminology of the first domain, requesting more emphasis on the fact that risks are not exclusively associated with prodromal phases of diseases but can also be linked to symptoms. Analogously, for *Detecting Timely Deterioration*, concerns were expressed on the definition that originally included an “increased sampling frequency of the clinical assessments.” This wording was considered inadequate and potentially misleading due to its overlap with the electronic interpretation of the concept of “sampling frequency.”

Given the results achieved in Round 2, three items were retained for subsequent voting: the domain definitions for *Predicting Risk* and *Timely Detecting Deterioration* and the research question for *Enhancing Diagnostic Procedure*.

### Round 3 results

Round 3 had a consistent response rate of 67% which was comparable to that of the previous round. The framework structure remained stable, with three categories and six underlying domains (Fig. S4a) confirming its robustness. Among the three retained items, experts reached a full consensus on all points. In addition to the consensus items, panel experts provided supplementary suggestions, including aligning the wording of definition and the research question for *Enhancing Diagnostic Procedure*.

**Fig. 3:**
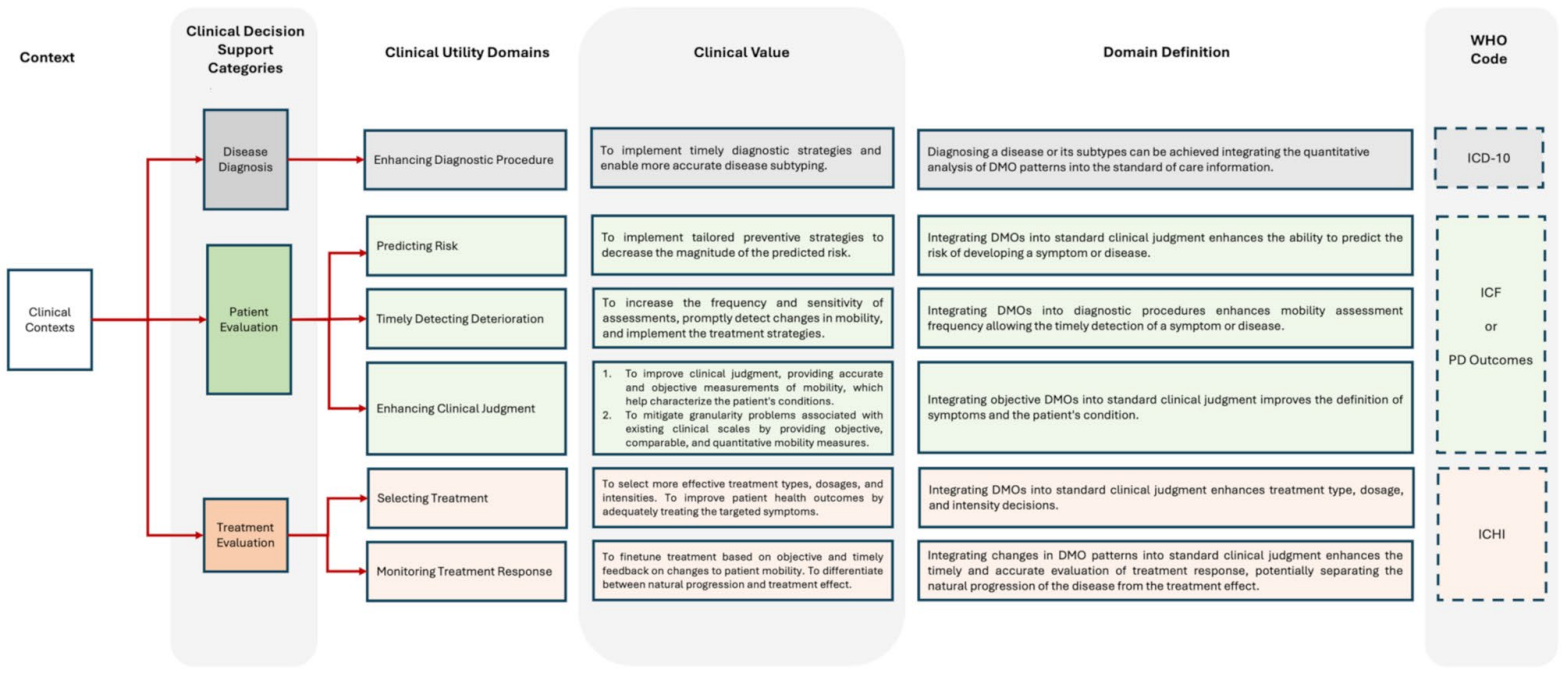
Final Clinical Utility Framework. The clinical contexts are divided into three main categories which align with internationally recognized existing clinical frameworks used by the world health organization. WHO World Health Organization, ICD-10: International Classification of Disease 10th revision. ICF: International Classification of Functioning, Disability and Health coding system. ICHI: International Classification of Health Interventions

Expert-level agreement was achieved on a final clinical utility framework composed of three categories and six underlying domains (Fig. 3). Full consensus was also reached on the overall framework concept, taxonomy, and domain definitions necessary, confirming the role of DMOs in personalized care in PD (Table 3).

**Table 3:** Framework taxonomy evolution by round. The table shows the evolution in terms of taxonomy of the different domains and categories according to the feedback from the expert panel

Framework Taxonomy Evolution					
Round 1		Round 2		Round 3	
Categories	Domains	Categories	Domains	Categories	Domains
-	-	Disease Diagnosis	Enhancing Diagnostic Procedure	Disease Diagnosis	Enhancing Diagnostic Procedure
Patient Evaluation	Risk Patterns Forecast	Patient Evaluation	Predicting Risk	Patient Evaluation	Predicting Risk
	Timely Diagnosis		Timely Detecting Deterioration		Timely Detecting Deterioration
	Enhancing Diagnostic Procedure		Enhancing Clinical Judgment		Enhancing Clinical Judgment
Treatment Evaluation	Individualized Intervention	Treatment Evaluation	Selecting Treatment	Treatment Evaluation	Selecting Treatment
	Treatment Monitoring		Monitoring Treatment Response		Monitoring Treatment Response

### Secondary study results

Throughout the study, the experts did not consider any domains currently applicable in clinical practice, with all the domains voted on average as “*1–5 years*” away from clinical utility (Table S5). Overwhelmingly, the most significant challenge to clinical applicability was the need for reference values, followed by concerns about the interpretability of DMOs (Table S6). Technology readiness was the main obstacle to implementing DMOs within clinical practice, followed closely by the cost associated with the technologies employed (Table S7). The latter stems from the limited information provided by gait as a single motor manifestation and difficulties in meaningfully visualizing the DMO-derived information. Finally, when asked about the role of DMOs, the experts considered them complementary rather than primary data sources useful for making or verifying a clinical decision (Fig. S8).

## Discussion

In this expert consensus manuscript, we developed a conceptual framework to bridge the gap between DMOs for research and DMOs for personalized clinical decision support. Using PD as a use case, we outlined specific clinical utility domains that can benefit from knowledge generated by DMOs.

A full consensus on a final clinical utility framework was achieved based on the feedback of twelve Mobilise-D multidisciplinary experts from six international institutions. This consisted of three main categories (*Disease Diagnosis*, *Patient Evaluation*, and *Treatment Evaluation*) and six underlying domains (*Enhancing Diagnostic Procedure*, *Predicting Risk*, *Timely Detecting Deterioration*, *Enhancing Clinical Judgment*, *Selecting Treatment*, and *Monitoring Treatment Response*). The framework ultimately defines three areas of application: identification of the disease, characterization of the patient, and support for treatment decisions.

The initial framework, comprised of only two categories (*Patient Evaluation* and *Treatment Evaluation*) and five domains, achieved consensus within Round 1, validating the appropriateness of the “treatment cycle” concept as a foundation for our work [29]. However, based on expert feedback, a new *Disease Diagnosis* category stemmed from the *Patient Evaluation* branch. The rationale was the conceptual distinction between performing the diagnostic workup (identifying a new disease or disease subtype) and evaluating progression (symptomatic/syndromic workups) within the context of an existing disease [30, 31]. This change closely aligns with clinical practice by emphasizing the clinical utility of DMOs in accurately classifying diseases via the International Classification of Disease (ICD) code [32, 33] or determining a change in the functional and disability health domains structured within the International Classification of Functioning, Disability and Health (ICF) coding system [34]. This alignment extends to the *Treatment Evaluation* branch, where the treatment selection domain allows for reporting health interventions via the International Classification of Health Interventions (ICHI) structure [35].

Although experts reached a consensus according to the double threshold rule, some concerns remained. A critical area of disagreement was associated with the feasibility of using DMOs to provide individualized interventions and treatment monitoring. Specifically, experts questioned the level of granularity provided by DMOs, with one expert stating that it appears impossible to detect monthly impairment changes at the single-patient level due to the extreme heterogeneity of PD clinical presentations. A second point of divergence was the prognostic power of DMOs. Some experts consider these digital biomarkers unable to provide any insight into future progression, as their information is limited to a snapshot of the present time.

These concerns were reflected in the answers related to the “Time to Applicability” rather than in the elements of the framework (Table S5). Nevertheless, it is essential to note that only a few experts considered DMOs to be immediately applicable in generating clinical utility, with most agreeing technological implementation is between 1 and 5 years from applicability. Interestingly, even if DMOs can generate clinical utility, the panel unanimously concluded that DMOs do not represent the primary source for making a clinical decision. Thus, experts agreed that DMOs play a key secondary, complementary role in the decision process or in verifying its outcomes.

A core objective of this framework is to facilitate the translation of the research knowledge of DMOs (i.e., symptomatic characterization and outcomes generated in research clinical trials) into clinical utility applicable to usual care. The latter can support clinical decisions through digital health information, which can be integrated as objective measures into diagnostic digital medical devices to tailor individualized interventions based on a patient’s profile. Additionally, this new information can complement the patient’s perspective and self-awareness of their symptoms and clinical experiences, thus directly supporting personalized care not only from the healthcare professional perspective but also from the patient’s side [36].

However, to achieve these goals, crucial challenges must be overcome to integrate DMOs into digital medical devices (e.g., FDA/EMA-certified tools) that can provide clinical decision support. Expert-derived feedback revealed that DMO interpretability, appropriate reference data, and meaningful visualizations for decision-making are the primary challenges in achieving clinical utility. These results align with what was previously reported by Sharma et al. [37] and complement the cost and data accuracy elements presented by Ambrens et al. [6]. Additionally, differences across medical procedures, heterogeneous reimbursement strategies, diverse levels of physician digital health literacy, are all aspects that increase the complexity of making DMOs applicable across different clinical settings and healthcare ecosystems and align with similar challenges reported throughout interdisciplinary care in PD [38]. For this reason, a significant amount of effort is still required in clinical research to translate DMOs into real-world clinical tools. This effort should come in parallel with continuous harmonization and update of the regulatory apparatus by national authorities [39].

It is essential to note that our framework is not yet complete. Its six clinical utility domains can guide future clinical studies seeking to implement DMOs within patient treatment cycles for personalized care. The individual research questions and results of such clinical studies will play a vital role in generating an additional layer of “subdomains” in our framework, where more precise, practical medical applications, contexts, and interpretations of DMOs’ values will be described. An example of a potential subdomain associated with our *Treatment Evaluation* category, and more specifically the *Selecting Treatment* branch, is the study of Somerset et al., where DMOs have successfully been utilized to tailor the deep brain stimulation parameters of patients with PD [40].

This study has some potential methodological limitations. First, the large dropout between the first and the second round, from 12 to 8 experts, could have partially biased the consensus results by reducing the amount of feedback received. Second, the electronic nature reduced the possibility of providing real-time feedback and requesting immediate clarification if the feedback was unclear. Additionally, it prevented experts from building upon ideas from fellow panel members. Third, the association between the panel experts and the longitudinal Mobilise-D clinical validation study [10] in parallel with the unbalance between clinical and technical backgrounds (10 vs. 2, see Table 1 for additionally details) may bias their perspective toward the applicability and clinical utility of the DMOs, as described in the paradox of choice. It is important to underline, however, that clinical experts involved in this study still present robust technical skills, crafted through participation in numerous top quality DMOs analysis. Furthermore, the iterative workflow increased confidence in the objectivity of the expert feedback nevertheless, future studies are required to validate our findings with larger and more heterogenous expert cohorts. Fourth, the small, discipline-unbalanced expert panel may limit the generalizability of the consensus findings, suggesting that future studies should involve broader expert groups across healthcare professions to ensure optimal identification of translational challenges and clinical applications that have not been considered by this work. However, our study achieved an average participation of 75%, with the number of rounds and participation rate consistent with those of other consensus-building studies [6, 20, 25]. Finally, our framework focuses exclusively on the clinical aspects of clinical utility. Further studies should investigate the legal, ethical, and regulatory dimensions influencing and governing the translation from research utility to clinical utility.

## Conclusions

Through a structured consensus-building process, we developed the first conceptual framework defining clinical utility domains where objective DMOs could support personalized care in PD. Experts reached a consensus on clinical utility domains aligned with disease diagnosis, patient evaluation, and treatment management, underscoring areas where DMOs have the potential to generate clinical value for the patient and the clinician.

From a consensus perspective, despite the study’s limitations, this framework provides a preliminary structure for subsequent studies to confirm our results with diverse expert cohorts and expand our findings beyond PD. From a DMOs clinical utility point of view, our results support researchers planning future clinical trials with contexts where DMOs can play a valuable role for clinical decision support in the management of PD, and insights on challenges associated with them. Ultimately, this is the first step toward developing guidelines to assess DMOs’ clinical utility and support their integration into Real World clinical practice.

## Supplementary Information

Below is the link to the electronic supplementary material.

Supplementary Material 1

## Data Availability

The dataset supporting the conclusions of this article is available in the repository: 10.17881/g865-hf21.

## Abbreviations

PD: Parkinson’s disease
DMOs: Digital Mobility Outcomes
ICD: International Classification of Disease
ICF: International Classification of Functioning, Disability and Health
ICHI: International Classification of Health Interventions
