# Supplementary material for: Consensus on the clinical utility of digital mobility outcomes for personalized clinical decision support in parkinson’s disease
Source: Neurol Res Pract. 2025 Sep 18;7(1):66. doi: 10.1186/s42466-025-00426-8 (PMC12447593; doi:10.1186/s42466-025-00426-8)

**Supplementary Material**

**Title:** Consensus on the Clinical Utility of Digital Mobility Outcomes for Personalized Clinical Decision Support in Parkinson’s Disease.

**Authors**: Alan CASTRO MEJIA^1†^, Stefano SAPIENZA^1*†^, Ivana PACCOUD^1^, Lisa ALCOCK^3,6^, Philip BROWN^4^, Heiko GAßNER^5,12^, Heather HUNTER^4^, Walter MAETZLER^8^, Anat MIRELMAN^9,10,11^, Alice NIEUWBOER^13,14^, Martin REGENSBURGER^5^, Lynn ROCHESTER^3,4,6^, Sabine STALLFORTH^5^, Beatrix VEREIJKEN^7^, Alison YARNALL^3,4^, and Jochen KLUCKEN^1,2^

*1. Luxembourg Centre for Systems Biomedicine, University of Luxembourg, Esch-sur-Alzette, Luxembourg*

*2. Centre Hospitalier de Luxembourg, Luxembourg, Luxembourg*

*3. NIHR Newcastle Biomedical Research Centre, Newcastle University, Campus for Ageing and Vitality, Newcastle upon Tyne, United Kingdom*

*4. The Newcastle upon Tyne Hospitals NHS Foundation Trust, Newcastle upon Tyne, United Kingdom*

*5. Department of Molecular Neurology, Friedrich-Alexander-Universität Erlangen-Nürnberg, Erlangen, Germany*

*6. Translational and Clinical Research Institute, Faculty of Medical Sciences, Newcastle University, Newcastle upon Tyne, United Kingdom*

*7. Department of Neuromedicine and Movement Science, Norwegian University of Science and Technology, Trondheim, Norway*

*8. Department of Neurology, University Hospital Schleswig-Holstein and Kiel University, Kiel, Germany*

*9. Laboratory for Early Markers of Neurodegeneration (LEMON), Tel Aviv Sourasky Medical Center, Tel Aviv, Israel*

*10. School of Medicine & Health Sciences, Tel Aviv University, Tel Aviv, Israel*

*11. Sagol School of Neuroscience, Tel Aviv University, Tel Aviv, Israel*

*12. Fraunhofer Institute for Integrated Circuits IIS, Erlangen, Germany*

*13. Neurorehabilitation Research Group (eNRGy), Department of Rehabilitation Sciences, KU Leuven, Leuven, Vlaams-Brabant, Belgium*

*14. Leuven Brain Institute (LBI), Leuven, Belgium*

* Corresponding author

^†^ Co-primary authors

**Correspondence:**

Name: Stefano Sapienza PhD

Affiliation: Luxembourg Center for Systems Biomedicine, Digital Medicine Group

Email: stefano.sapienza@uni.lu

**SUPPLEMENTARY MATERIALS - TABLE OF CONTENTS**

[**S1.**Round 1 Voting Results for Framework Evaluation 3](#_Toc193980241)

[**S2.**Round 1 Voting Results for Domain Clinical Contexts 4](#_Toc193980242)

[**S3.**Round 1 Voting Results for Domain Clinical Values 5](#_Toc193980243)

[**S4.**Round 2 New Domain (a) and Updated (b) Framework Structures 6](#_Toc193980244)

[**S5.** Secondary: Results for Domain Time to Applicability 7](#_Toc193980245)

[**S6.** Secondary: Results for Foreseen Challenges in Clinical Applicability 8](#_Toc193980246)

[**S7.** Secondary: Main obstacle to the implementation of the DMOs within clinical practice. 9](#_Toc193980247)

[**S8.** Secondary: Expert opinion on the main role of DMOs for clinical decisions. 10](#_Toc193980248)

#### **S1.**Round 1 Voting Results for Framework Evaluation

| **Framework** | **Strongly agree** | **Agree** | **Neither agree or disagree** | **Disagree** | **Strongly disagree** | **Average Agreement** |
| --- | --- | --- | --- | --- | --- | --- |
| **Risk Pattern Forecast** | 54.55 | 27.27 | 9.09 | 9.09 | 0.00 | 4.27 |
| **Timely Diagnosis** | 54.55 | 45.45 | 0.00 | 0.00 | 0.00 | 4.55 |
| **Enhancing Diagnostic Power** | 54.55 | 27.27 | 9.09 | 9.09 | 0.00 | 4.27 |
| **Individualized Intervention** | 63.64 | 36.36 | 0.00 | 0.00 | 0.00 | 4.64 |
| **Treatment Monitoring** | 63.64 | 27.27 | 0.00 | 9.09 | 0.00 | 4.45 |

#### **S2.**Round 1 Voting Results for Domain Clinical Contexts

| **Clinical Contexts** | **Strongly agree** | **Agree** | **Neither agree or disagree** | **Disagree** | **Strongly disagree** | **Average Agreement** |
| --- | --- | --- | --- | --- | --- | --- |
| Risk Pattern Forecast | 54.55 | 27.27 | 18.18 | 0.00 | 0.00 | 4.36 |
| Timely Diagnosis | 81.82 | 18.18 | 0.00 | 0.00 | 0.00 | 4.82 |
| Enhancing Diagnostic Power | 54.55 | 27.27 | 27.27 | 0.00 | 0.00 | 4.36 |
| Individualized Intervention | 45.45 | 27.27 | 9.09 | 18.18 | 0.00 | 4.00 |
| Treatment Monitoring | 45.45 | 45.45 | 0.00 | 9.09 | 0.00 | 4.27 |

#### **S3.**Round 1 Voting Results for Domain Clinical Values

| **Clinical**  **Value** | **Strongly agree** | **Agree** | **Neither agree or disagree** | **Disagree** | **Strongly disagree** | **Average Agreement** |
| --- | --- | --- | --- | --- | --- | --- |
| Risk Pattern Forecast | 36.36 | 36.36 | 27.27 | 0.00 | 0.00 | 4.09 |
| Timely Diagnosis | 54.55 | 36.36 | 9.09 | 0.00 | 0.00 | 4.45 |
| Enhancing Diagnostic Power | 45.45 | 27.27 | 18.18 | 9.09 | 0.00 | 4.09 |
| Individualized Intervention | 27.27 | 45.45 | 9.09 | 9.09 | 9.09 | 3.73 |
| Treatment Monitoring | 45.45 | 36.36 | 9.09 | 9.09 | 0.00 | 4.18 |

#### **S4.**Round 2 New Domain (a) and Updated (b) Framework Structures


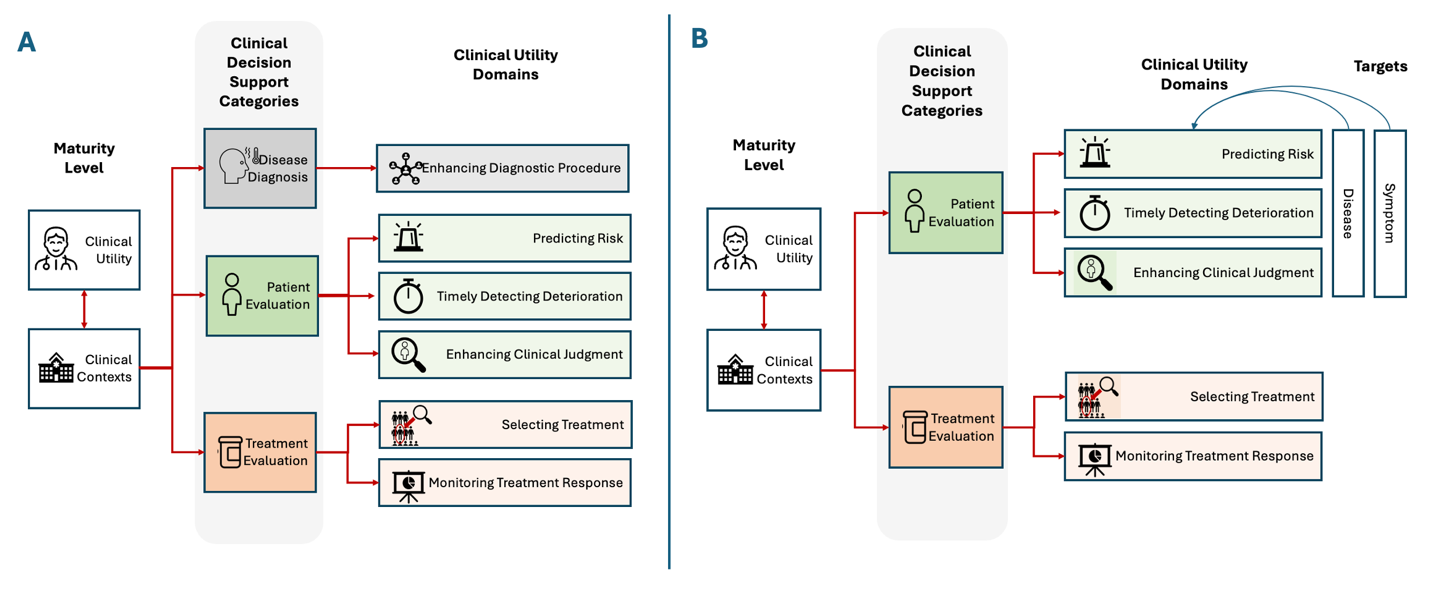


Notes:  .

#### **S5.** Secondary: Results for Domain Time to Applicability

| **Timeline to Applicability** | **Don’t know** | **Now** | **1-5 years** | **5-10 years** | **>10 years** | **Not Clinically Relevant Applicable** | **Most Selected Option** |
| --- | --- | --- | --- | --- | --- | --- | --- |
| Risk Patterns Forecast | 0.00 | 9.09 | **54.55** | 27.27 | 9.09 | 0.00 | In 1 to 5 years |
| Timely Diagnosis | 0.00 | 9.09 | **81.82** | 0.00 | 9.09 | 0.00 | In 1 to 5 years |
| Enhancing Diagnostic Procedure | 4.55 | 4.55 | **59.10** | 22.73 | 9.09 | 0.00 | In 1 to 5 years |
| Individualized Intervention | 3.03 | 9.09 | **39.39** | 36.36 | 9.09 | 3.03 | In 1 to 5 years |
| Treatment Monitoring | 4.55 | 13.64 | **50** | 18.18 | 13.64 | 0.00 | In 1 to 5 years |

**Note:** Table denotes relative distribution and average agreement of expert feedback responses on the different example applicability timeline

#### **S6.** Secondary: Results for Foreseen Challenges in Clinical Applicability

**
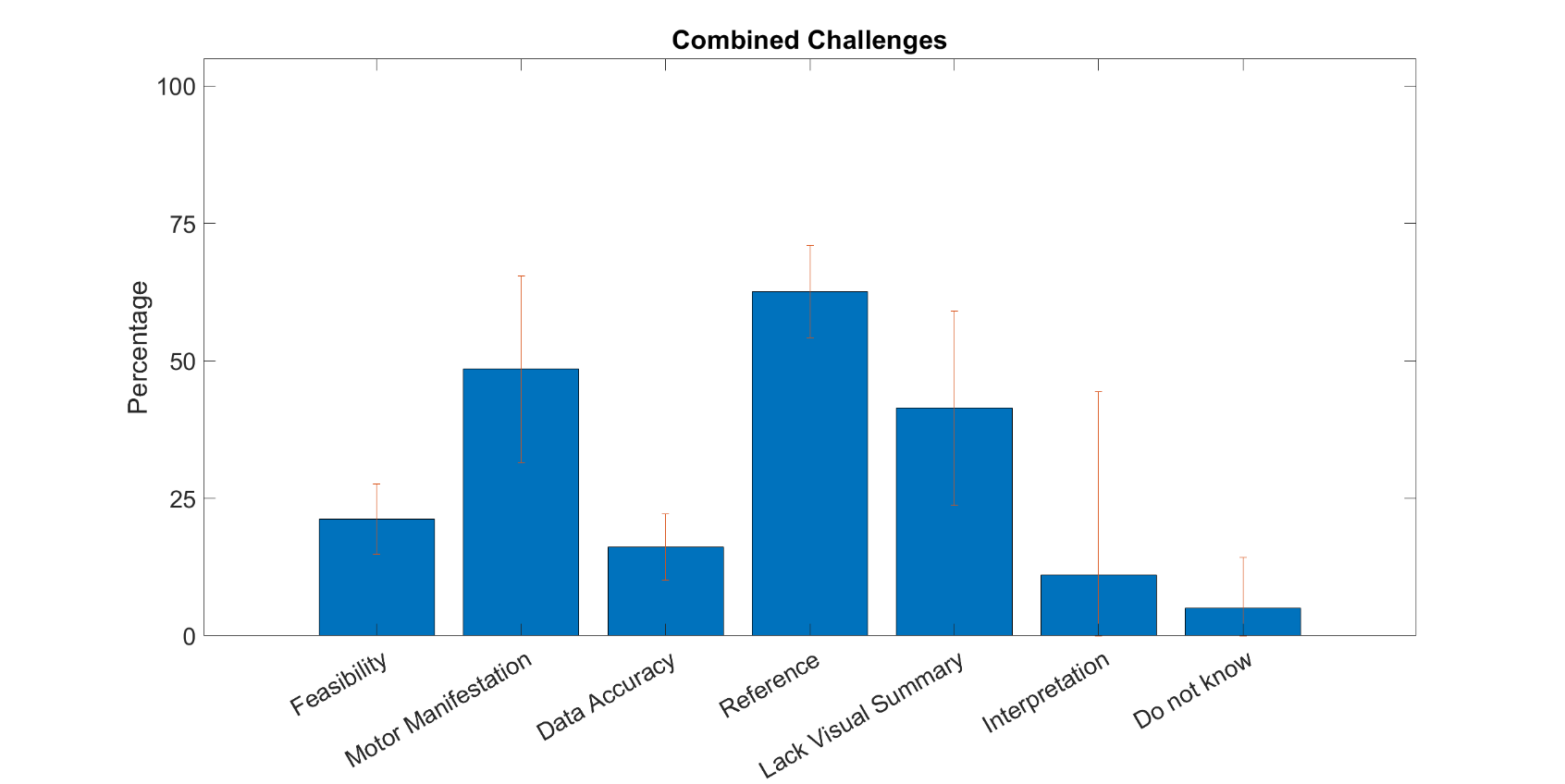
**

**Note:** Aggregated results from the challenges foreseen. Error bars represent the standard deviation of the occurrence of the item across different examples.

#### **S7.** Secondary: Main obstacle to the implementation of the DMOs within clinical practice.


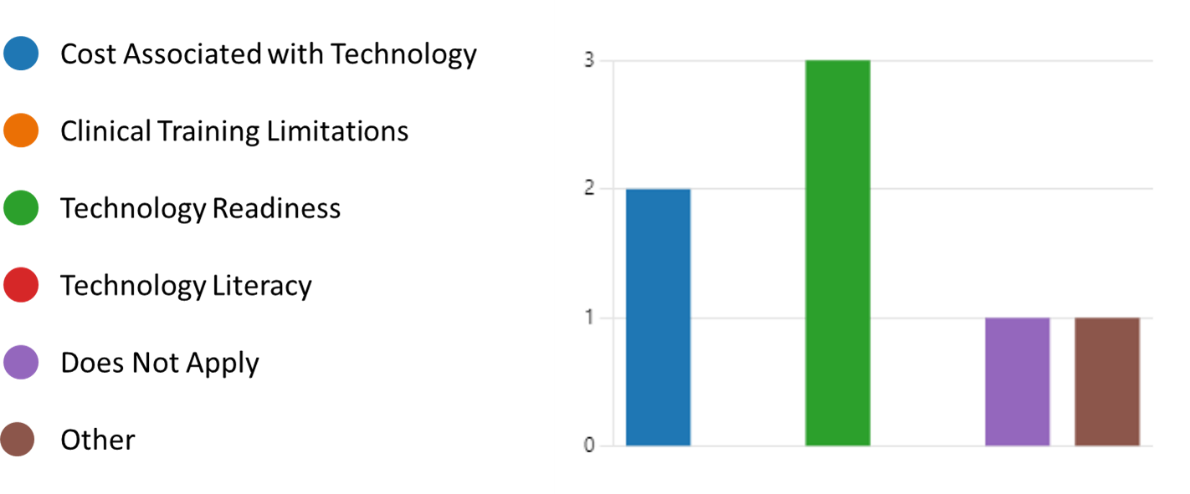


#### **S8.** Secondary: Expert opinion on the main role of DMOs for clinical decisions.


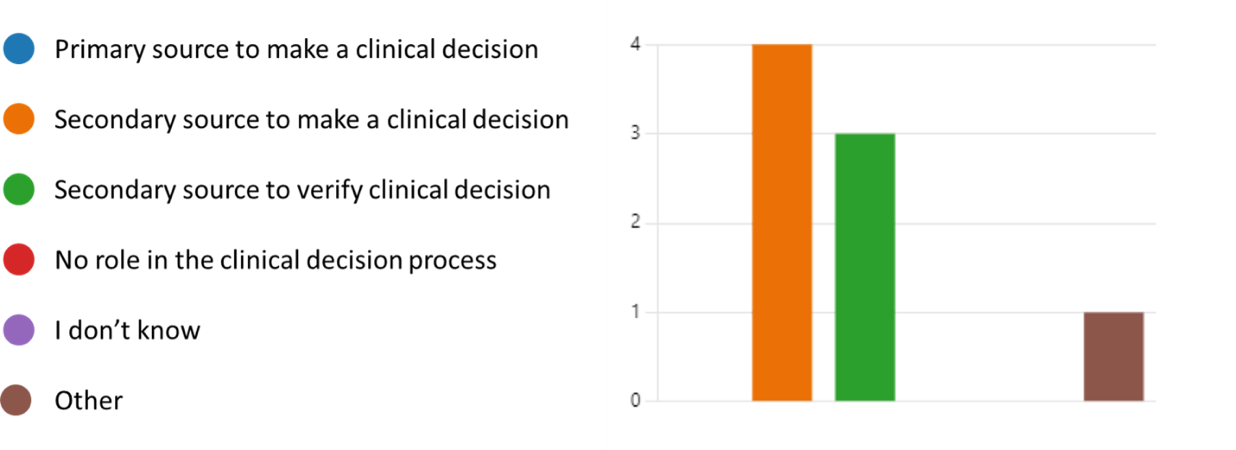

Supplement: Supplementary file 1 — Supplementary Material 1 [file 42466_2025_426_MOESM1_ESM.docx]
